# The spectrum of square wave jerks in essential tremor: an exploratory study

**DOI:** 10.1007/s00702-026-03134-y

**Published:** 2026-03-17

**Authors:** Claudio Terravecchia, Roberta Terranova, Alessandra Rufa, Aasef G. Shaikh, Clara Grazia Chisari, Andrea Salerno, Giulia Donzuso, Calogero Edoardo Cicero, Giovanni Mostile, Mario Zappia, Alessandra Nicoletti

**Affiliations:** 1 Department “G.F. Ingrassia”, Section of Neurosciences, University of Catania, Via Santa Sofia 78, Catania, 95123 Italy; 2 Eye tracking and Visual Application Lab (EVA Lab), Department of Medicine, Surgery and Neurosciences, University of Siena, Siena, 53100 Italy; 3 University Hospitals and Cleveland VA Medical Center, Case Western Reserve University, Cleveland, OH USA; 4 Oasi Research Institute - IRCCS, Troina, Italy

**Keywords:** Essential tremor, Eye movements, Saccadic intrusions, Square wave jerks, Saccades

## Abstract

Square wave jerks (SWJ) are saccadic intrusions interrupting fixation, reflecting an inhibitory deficit linked to impaired cerebellar-brainstem-basal ganglia networks. Cerebellar pathways including fastigial nucleus exert a central role in determining both SWJ subtypes and saccadic adaptation. We aimed to investigate eye movements abnormalities in Essential Tremor (ET) with a particular focus on SWJ subtypes, assessing their possible role in disease characterization. Different SWJ subtypes during primary position fixation task as well as visually-guided saccades (VGS) were evaluated among healthy controls (HCs), pure ET and ET-plus patients through Eyelink 1000-Plus. Forty tremor patients [10 pure ET (25%) and 30 ET-plus (75%)] and 23 HCs were enrolled. Adjusting by age, higher rate of biphasic SWJ (BSWJ) was found in tremor population compared to HCs. The rate of BSWJ was even higher in pure ET as compared to ET-plus. A new pattern of “triphasic” SWJs as well as “staircase” SWJ were found in pure ET and ET-plus but not in HCs. Higher variability on horizontal VGS parameters was found in tremor patients compared to HCs and a trendwise increase was also seen across HCs, ET-plus and pure ET. Fixation and saccadic oculomotor abnormalities were demonstrated in ET patients with clinical-instrumental correlates, showing the possible usefulness of quantitative eye movement assessment in ET characterization.

**Supplementary Information:**

The online version contains supplementary material available at https://doi.org/10.1007/s00702-026-03134-y.

## Introduction

In the last decades the clinical phenotype of essential tremor (ET) has been expanded from a “benign”, monosymptomatic condition mainly characterized by *“bilateral*,* largely symmetric postural or kinetic tremor involving hands and forearms”* (Deuschl et al. 1998) to a broadly heterogeneous neurodegenerative disease encompassing a variety of both motor and non-motor symptoms (Louis et al. 2021). In order to better characterize this phenotypical heterogeneity, a new clinical classification attempt has been proposed, recognizing ET as a syndrome rather than a single homogeneous nosological entity (Bhatia et al. 2018). Moreover, the distinction between pure “*ET*” and “*ET-plus*” (ET-P) has been introduced (Bhatia et al. 2018), which quickly raised several controversies mainly due to the lack of reliable clinical and neurophysiological supportive evidences (Louis et al. 2020). On this account, objective instrumental biomarkers may represent valuable tools to further characterize ET (Mostile et al. 2021; Terravecchia et al. 2024a; Terranova et al. 2025).

Several evidences suggest a strong relation between ET and cerebellar dysfunction involving the cerebello-thalamo-cortical pathways (Louis 2016; Holtbernd et al. 2021). On the other hand, cerebellum also exerts a pivotal role in saccadic and fixation oculomotor control (Leigh et al. 2015; Optican et al. 1980; Takagi et al. 1998; Otero-Millan et al. 2011; Otero-Millan et al. 2013). In particular, the cerebellar oculomotor vermis/fastigial oculomotor region (OMV/FOR) is involved in saccadic accuracy trough an adaptation process of error detection and subsequent correction (Optican et al. 1980, Takagi et al. 1998). FOR is also involved in mechanisms underlying a stable fixation (Leigh et al.2015, Otero-Millan et al. 2011; Otero-Millan et al. 2013). Indeed, involuntary saccades interrupting fixations may be observed in several disorders involving cerebellar-brainstem-basal ganglia networks (Otero-Millan et al. 2013). Among saccadic intrusions, the most common type is represented by square wave jerks (SWJ), especially in their monophasic morphology (MSWJ) consisting in conjugate couplets of horizontal back-to-back saccades interrupted by an intersaccadic interval (Abadi et al. 2004; Lemos et al. 2013). However, other SWJ subtypes mainly differing in their eye movement pattern allowing the gaze to return in primary fixation position were also described (Lemos et al. 2013, Shaikh et al. 2011). From a pathophysiological point of view, the first intrusive saccadic movement may result from a lacking inhibition acting on superior colliculus (SC) or directly on brainstem burst neurons during fixation, leading to a positional error which triggers a corrective saccade through FOR-mediated mechanisms (Otero-Millan et al. 2011, 2013). Thus, a cerebellar dysfunction affecting saccadic accuracy may also impair the corrective SWJ movements, influencing SWJ morphology.

On this light, a detailed analysis of different SWJ subtypes coupled with a standardized visually-guided saccades (VGS) assessment may provide useful insights in a strongly cerebellar-related disorder such as ET. In particular, we hypothesized a possible association between ET and higher fixation instability (i.e. higher SWJ rates, especially with complex morphologies) as well as higher saccadic variability, reflecting cerebellar dysfunction. On the other hand, several evidences point to adjunctive pathophysiological mechanisms beyond cerebellar dysfunction (Parida et al. 2024; Paparella et al. 2024), especially in ET-P which encompasses several clinical extra-cerebellar “soft signs” (i.e. rest tremor, questionable dystonia). In this context, a prominent cerebellar dysfunction could be considered in pure ET in respect with ET-P. On this light, how possibly cerebellar-related fixation and saccadic abnormalities could be differently expressed in these two subgroups of patients also needs to be investigated.

To date, very few studies investigated eye movements in ET showing quite heterogeneous results (Helmchen et al. 2003; Visser et al. 2019; Gitchel et al. 2013; Wójcik-Pędziwiatr et al. 2016; Rekik et al. 2023), while a detailed study of fixation abnormalities has never been performed. We aimed to address these points in order to assess possible clinical-instrumental correlations as well as their potential role in ET characterization.

## Materials and methods

### Study population

Subjects attending the *Parkinson’s Disease and Movement Disorders Centre* at the University of Catania, Italy, fulfilling the diagnostic criteria for pure “*ET*” and *“ET-Plus”* (ET-P) (Bhatia et al. 2018) as well as a group of healthy controls (HCs) among the patients’ caregivers were consecutively enrolled.

All subjects underwent a standardized neurological examination performed by a neurologist expert in movement disorders as well as instrumental oculomotor assessment, provided in their diagnostic workup. Motor impairment was assessed by TETRAS Scale (Elble et al. 2012). Study protocol was approved by Local Ethics Committee. Written informed consents were obtained from study subjects.

### Eye movements recording protocol

Binocular eye movements were recorded at 1000 Hz frequency using a video-based eye tracking system (Eyelink 1000 Plus, SR Research). A 544 × 306 mm LCD display, with a resolution of 1920 × 1080 pixels, positioned 660 mm from the subject was used. Head movements were minimized by a headrest. The stimulus was a black dot with a diameter of 0.4 deg on a white background. SWJ were evaluated during a 60-seconds central position fixation task. Concerning VGS protocol, subjects were instructed to fixate on the target. It started in a central position, then, after a variable interval (900–1200 ms), it switched off and simultaneously reappeared at a pseudo-random position resulting from a selection among predefined amplitudes in horizontal and vertical plane, at 10 or 15 and 5 or 8 degrees, respectively. Different directions were uniformly distributed. A total of 40 horizontal and 40 vertical trials were recorded for each subject.

### Eye movements data processing protocol

**Fig. 1:**
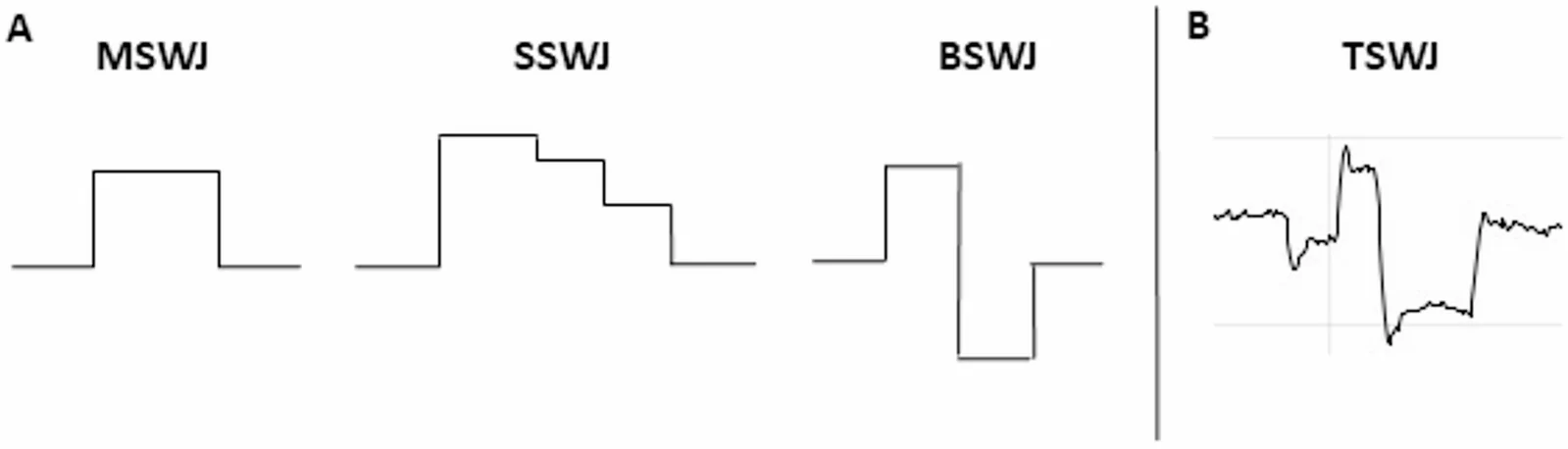
SWJ patterns investigated in the study. **A** Monophasic, “Staircase” and Biphasic SWJ patterns. **B** Identified Triphasic SWJ pattern in tremor population

Data processing was performed off-line using the Eyelink Data Viewer Software (SR Research Ltd). Saccade onset/ending was defined by a velocity threshold of 30 deg/s. MSWJ, Biphasic SWJ (BSWJ), Staircase SWJ (SSWJ) (Fig. 1A) and any other SWJ morphologies were assessed (Abadi et al. 2004). For each SWJ subtype, its rate/min and the following parameters were evaluated: peak and mean velocity, amplitude, duration and intersaccadic intervals. Relevant VGS parameters were also assessed: peak and mean velocity, amplitude, latency, duration and gain (primary saccade amplitude divided by target amplitude) (Terravecchia et al. 2024b). For each parameter its variation coefficient (VC) was calculated as the ratio between its standard deviation and mean value among the subject’s trials. The main sequence linear models were applied to examine the relations between saccadic amplitude (independent variable) and its peak velocity and duration (dependent variables) (Gibaldi et al. 2021).

### Statistical analysis

Data were analyzed using STATA 18. Quantitative variables were described using mean and standard deviation. Data normality was tested by Shapiro-Wilk test. Groups difference between means was evaluated by independent-samples t-test or Wilcoxon rank-sum test, while the difference between proportions by chi-squared test. Multivariate logistic regression analysis was performed and parameters associated with the outcome at the univariate analysis with a threshold of *p* = 0.10 were included in the model as well as age (possible a priori confounder). For oculomotor parameters significantly differing between total tremor population and HCs, correlation analysis with tremor severity (i.e. TETRAS Performance Score – TETRAS-PS) were assessed by Spearman’s rank correlation. A multivariate linear regression analysis was also performed considering age as a possible a priori confounder.

The Chow test was applied to test equality of two independent linear regression main sequence models.

The tremor population was investigated both as a whole and further stratifying by pure ET and ET-plus (ET-P). Possible differences in oculomotor parameters based on the presence/absence of single clinical “soft signs” were also explored. Jonckheere–Terpstra test was used to assess possible trends in relevant instrumental parameters (dependent variables) across groups (independent variable), sorted in ascending order: HCs, ET-P and pure ET. This ordinal structure was defined considering a prominent cerebellar dysfunction in pure ET in respect with ET-P, in which a variety of extra-cerebellar “soft signs” are included. A multivariate linear regression analysis was also performed considering age as a possible a priori confounder. Linear regression assumptions (i.e. absence of multicollinearity, homoscedasticity and residual normality) were tested. In particular, variance inflaction factor (VIF) was calculated to exclude multicollinearity (i.e. VIF > 5). Moreover, an information matrix test on the linear regression model, and then Cameron & Trivedi’s orthogonal decomposition for heteroscedasticity, skewness, and kurtosis tests were applied. The White test for unrestricted homoscedasticity was also performed. When appropriate, the robust (or Huber-White/sandwich) estimator of variance was applied to increase inference reliability by allowing for valid statistical testing. Significance level was set at *p* < 0.05.

## Results

Forty tremor patients [28 men (70.0%); mean age ± SD: 71.6 ± 5.8; *N* = 10 ET (25%), *N* = 30 ET-P (75%)] and 23 HCs [12 men (52.2%); mean age ± SD: 65.4 ± 7.3] were enrolled (Table 1). A statistically significant lower age was found in HCs as compared to the tremor population (*p* < 0.001).

**Table 1:** Demographic and Clinical Characteristics of Tremor Population

	Tremor population *N* = 40	Pure ET *N* = 10 (25.0%)	ET-Plus *N* = 30 (75.0%)	Pure ET vs. ET-Plus *p*-value
Age	71.6 ± 5.8	71.0 ± 7.4	71.9 ± 5.3	0.688
Males (%)	28 (70.0)	6 (60.0)	22 (73.3)	0.426
Soft signs (%)				
Rest tremor (%)	18 (45.0)	/	18 (60.0)	/
Questionable dystonia (%)	16 (40.0)	/	16 (53.3)	/
Impaired tandem gait (%)	2 (5.0)	/	2 (6.7)	/
Age at onset	51.1 ± 22.1	59.8 ± 16.5	48.2 ± 23.2	0.218
Disease duration	20.6 ± 19.1	11.3 ± 11.4	23.7 ± 20.2	0.116
Tremor medications (%)	13 (32.5)	3 (30.0)	10 (33.3)	0.845
Propranolol (%)	11 (27.5)	3 (30.0)	8 (66.7)	0.838
Topiramate (%)	1 (2.5)	0	1 (3.3)	0.559
Primidone (%)	1 (2.5)	0	1 (3.3)	0.559
TETRAS ADL	14.0 ± 10.3	9.0 ± 6.6	15.5 ± 10.8	0.127
TETRAS PS total	18.0 ± 6.0	14.7 ± 4.5	19.5 ± 6.1	0.033*

*: *p* < 0.050

No differences on age and sex distribution were reported between ET and ET-P while ET-P showed higher TETRAS–Performance Score compared to pure ET (Table 1).

**Fig. 2:**
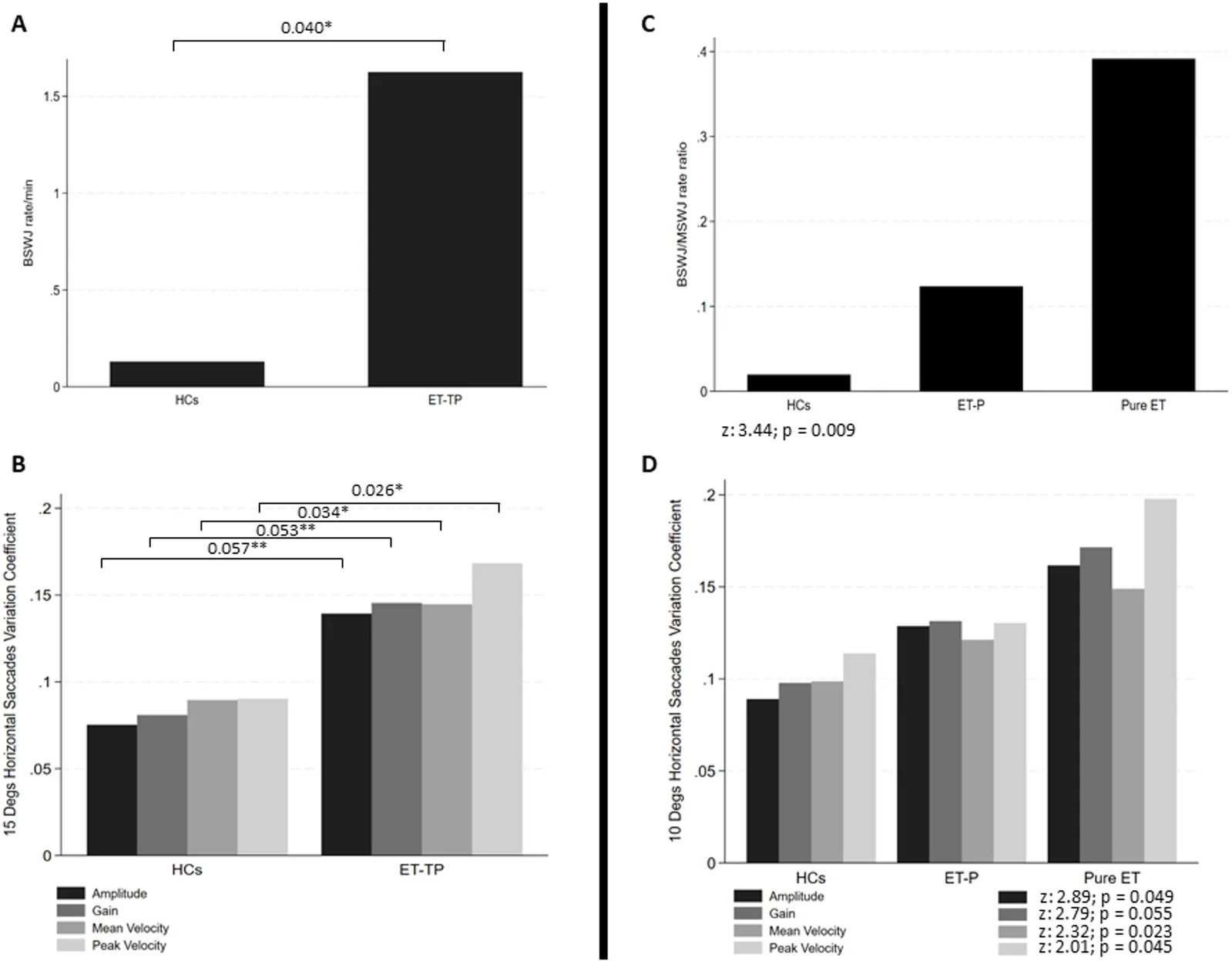
Significant differences and trends in SWJ patterns and VGS parameters across study groups. Significant differences in SWJ pattern (**A**) and VGS parameters (**B**) between HCs and tremor population (ET-TP); significant trends in SWJ patterns (**C**) and VGS parameters (**D**) across HCs, ET-P and pure ET populations. *: *p* < 0.050; **: *p* < 0.100

Concerning SWJ, a higher prevalence of subjects with SSWJ and BSWJ as well as higher total SWJ, MSWJ, SSWJ and BSWJ rate/min were found in tremor population compared to HCs (Table 2). Adjusting by age, a higher prevalence of subjects with BSWJ and a higher BSWJ rate/min were confirmed in tremor population compared with HCs (Table 2; Fig. 2A). A “triphasic” SWJ (TSWJ) pattern (Fig. 1B) was found in *N* = 5 tremor patients (12.5%) with a mean rate/min of 0.1 ± 0.6. Similarly, SSWJ pattern was found in tremor population (*N* = 8, 20.0%, mean rate/min: 0.2 ± 0.5) but not among HCs. No differences on SWJ kinematic parameters (i.e. amplitude, mean and peak velocity, duration, intersaccadic interval) were found between tremor population and HCs (Supplemental Tables S1–S4). A borderline-significant age-adjusted negative correlation between BSWJ rate and TETRAS-PS (rho: -0.30, *p* = 0.064) was found in total tremor population.

**Table 2:** Square wave jerks parameters across study groups

	HCs *N* = 23	Tremor population *N* = 40	*p*-value	Adj *p*-value^§^	Pure ET *N* = 10	ET-Plus *N* = 30	*p*-value	Adj *p*-value^§^
Subjects with SWJ (%)								
Total SWJ	17 (73.9)	36 (90.0)	0.093	0.343	8 (80.0)	28 (93.3)	0.224	0.245
MSWJ	17 (73.9)	36 (90.0)	0.093	0.343	8 (80.0)	28 (93.3)	0.224	0.245
SSWJ	/	8 (20.0)	0.022*	/	3 (30.0)	5 (16.7)	0.361	0.368
BSWJ	2 (9.1)	18 (45.0)	0.003*	0.015*	6 (60.0)	12 (40.0)	0.271	0.277
TSWJ	/	5 (12.5)	0.077	/	2 (20.0)	3 (10.0)	0.408	0.416
SWJ rate/min								
Total SWJ	5.1 ± 7.3	12.6 ± 2.3	0.008*	0.096	16.7 ± 18.6	11.2 ± 12.8	0.563	0.303
MSWJ	5.0 ± 12.7	10.5 ± 12.5	0.021*	0.174	12.7 ± 15.8	9.8 ± 11.5	0.823	0.531
SSWJ	/	0.2 ± 0.5	0.040*	/	0.4 ± 0.7	0.2 ± 0.4	0.345	0.198
BSWJ	0.1 ± 0.5	1.6 ± 2.3	0.001*	0.040*	3.2 ± 3.1	1.1 ± 1.8	0.071	0.023*
TSWJ	/	0.1 ± 0.6	0.187	/	0.5 ± 1.3	0.1 ± 0.4	0.400	0.227

^§^: adjusted by age; *: *p* < 0.050

Subsequent analysis was done after separating tremor in pure ET and ET-P. After adjusting by age, higher BSWJ rate/min was found in pure ET compared with ET-P (Table 2). No significant differences concerning SWJ were found based on the presence/absence of single clinical “soft signs”. Moreover, positive trends concerning the rates of total SWJ (z = 2.63, *p* = 0.066, borderline significant), SSWJ (z = 2.58, *p* = 0.045), BSWJ (z = 3.65, *p* = 0.002) as well as BSWJ/MSWJ rate ratios (z = 3.44, *p* = 0.009 were found across HCs, ET-P and pure ET groups, adjusting by age (Fig. 2C).

No differences on quantitative SWJ parameters were reported between ET and ET-P (Supplemental Tables S1–S4).

Concerning VGS, higher latencies, lower horizontal amplitude, velocities and gain as well as higher VC of horizontal parameters were found in tremor population compared to HCs at univariate analysis. Adjusting by age, higher VC of mean and peak velocity on horizontal 15-degrees VGS were confirmed in tremor compared to HCs, while borderline significant higher VC of amplitude (*p* = 0.064) and mean velocity (*p* = 0.099) on horizontal 10-degrees VGS as well as higher VC of amplitude (*p* = 0.057) and gain (*p* = 0.053) on horizontal 15-degrees VGS were found in tremor compared to HCs (Fig. 2B, Supplemental Table S5). No significant correlations between VGS parameters significantly differing from HCs (i.e. VC of mean and peak velocity on horizontal 15-degrees VGS) and TETRAS-PS were found in total tremor population.

Main sequence models did not differ between tremor and HCs (Supplemental Materials - Figure S1).

After adjusting by age, a higher VC of peak velocity on horizontal 10-degrees VGS was found in pure ET compared to ET-P (Supplemental Materials - Table S6). No significant differences concerning VGS parameters were found based on the presence/absence of single clinical “soft signs”.

Positive trends in VC of amplitude (z = 2.89, *p* = 0.049), gain (z = 2.79, *p* = 0.055, borderline significant), peak and mean velocity (z = 2.01, *p* = 0.004; z = 2.32, *p* = 0.023, respectively) on horizontal 10-degrees VGS as well as in VC of peak and mean velocity (z = 3.21, *p* = 0.004; z = 3.17, *p* = 0.005, respectively) on horizontal 15-degrees VGS were found across HCs, ET-P and pure ET, adjusting by age (Fig. 2D).

## Discussion

In the present study eye movements abnormalities in ET were assessed to explore their possible role in disease characterization, with a particular focus on fixation disorders. A higher BSWJ rate and an increased variability on horizontal VGS parameters were found in tremor population compared to HCs. Moreover, SSWJ and TSWJ were recognized in tremor but not in HCs. Notably, a higher BSWJ rate as well as an increased horizontal VGS peak velocity variability was demonstrated in pure ET compared to ET-P. Finally, positive trends on SWJ rates, especially BSWJ, as well as on horizontal VGS parameters’ variability were found across HCs, ET-P and pure ET.

To date few studies investigated eye movements in ET, showing heterogeneous results. In particular, two studies previously assessed MSWJ in ET (Gitchel et al. 2013; Rekik et al. 2023) showing an increased MSWJ rate compared to HCs. Concerning VGS, some previous investigations reported no differences between ET and HCs (Helmchen et al. 2003; Visser et al. 2019), while reduced peak velocity (Gitchel et al. 2013), increased dysmetria (Wójcik-Pędziwiatr et al. 2016) and saccadic latency (Gitchel et al. 2013; Wójcik-Pędziwiatr et al. 2016) were reported elsewhere. When interpreting these findings, the great clinical and demographic heterogeneity in study populations as well as technical differences concerning recording protocols should be considered. Notably, none of these previous investigations accounted for the currently proposed distinction between pure ET and ET-P.

Concerning saccadic intrusions, as far as we know this is the first study specifically assessing different SWJ subtypes in ET, showing an increased BSWJ rate as well as the presence of SSWJ and a TSWJ pattern in tremor population compared to HCs. Differently from previous investigations, no significant differences concerning MSWJ were found between tremor and HCs after adjusting by age. Notably, MSWJ represents the most frequent SWJ pattern, commonly described also in elderly healthy subjects (Abadi et al. 2004; Lemos et al. 2013. Nevertheless, an increased MSWJ rate and amplitude has been recognized in several neurological diseases encompassing pakinsonian and cerebellar disorders (Otero-Millan et al. 2013; Sharpe et al. 1982; Rascol et al. 1991; Vidailhet et al. 1994).

Several structures are involved in SWJ pathophysiology. In particular, during fixation the SC may trigger saccadic inputs toward brainstem structures both as the result of spontaneous neural fluctuations and in response to fixation error signals (Otero-Millan et al. 2011). Thus, a random fluctuation in SC activity may cause the first SWJ-related saccade which in turn determines a fixation error, driving a further corrective movement (i.e. MSWJ) (Otero-Millan et al. 2013). Accordingly, SWJ may result from a “neural noise” on SC or brainstem saccade-generating circuits both in healthy subjects and in neurological diseases. Another possible mechanism generating SWJ may be related to an impaired control on brainstem burst neurons due to a cerebellar/FOR dysfunction. In this case, a dysmetric “corrective” saccade may also be triggered in response to the gaze position error induced by the first saccade, thus producing complex SWJ patterns (Otero-Millan et al. 2013). Indeed, SSWJ and “multiphasic” SWJ (i.e. BSWJ and TSWJ) may respectively represent the result of hypometric and hypermetric “corrective” responses to the former saccadic intrusive movement. On this light, our findings showing increased BSWJ rate as well as the presence of SSWJ and TSWJ in tremor population may further indicate a cerebellar functional involvement in ET, enlighting the possible role of SWJ morphology in disease characterization.

On the other hand, no significant differences on SWJ metrics have been found in tremor patients compared to HCs, in line with previous evidences on MSWJ (Gitchel et al. 2013).

Due to the relatively small sample size and the very low prevalence of other SWJ morphologies in our population, further considerations would be currently speculative. Larger studies are needed to further investigate possible clinical correlates of rarer SWJ morphologies (i.e. TSWJ and SSWJ) in order to better contextualize the current exploratory findings.

Concerning VGS, our data on VGS metrics showing no differences between tremor and HCs are at least partly in line with previous evidences (Helmchen et al. 2003; Visser et al. 2019). However, increased latencies and reduced horizontal VGS velocities and gain were found at univariate analysis in tremor compared to HCs, but not confirmed adjusting by age. Due to the relatively small study population we cannot entirely exclude a type II error.

On the other hand, the VGS parameters’ VC was firstly investigated, showing a significantly increased variability on horizontal VGS velocities and a borderline-significant increase on amplitudes and gain VC in tremor compared to HCs. A similar behavior was previously described as a proxy of saccadic inaccuracy in cerebellar syndromes resulting from possible OMV/FOR dysfunction (Federighi et al. 2011). Indeed, these structures allow saccadic adaptation through a process of error detection and subsequent correction acting both directly to saccadic burst generator (Scudder et al. 2003; Kojima et al. 2008) and indirectly to cortical areas through basal ganglia and thalamus. However, a concomitant impairment of saccadic metrics and dynamics was also demonstrated in cerebellar syndromes (Federighi et al. 2011). Thus, we may consider our findings as the expression of a possible milder mesial cerebellar dysfunction in ET. Nevertheless, an increased variability on saccadic gain and velocity was also described in Parkinson’s Disease (Nemanich et al. 2016). Thus, considering the great ET syndrome clinical heterogeneity, we cannot exclude a possible concomitant functional role of other brain pathways (i.e. basal ganglia) in determining our findings.

From a clinical perspective, it should be noted that higher BSWJ rate was found in pure ET compared to ET-P as well as a positive trend on BSWJ rate and horizontal VGS parameters’ variability across HCs, ET-P and pure ET. Several controversies have been raised concerning the concept of ET-P as an independent entity from pure ET or as a possible different state of the same disease (Louis et al. 2020). Moreover, ET-P represents a very heterogeneous population encompassing several clinical “soft signs” (i.e. rest tremor, questionable dystonia) with possible different pathophysiological substrates, in which context a less prominent cerebellar dysfunction could be hypothesized in respect with pure ET. Thus, taking also into account the relatively small sample size of our population, especially of the pure ET group, as well as the cross-sectional study design, we may consider our results as the possible expression of a more isolated cerebellar condition rather than a neurophysiological distinctive biomarker of pure ET in respect to ET-P.

On the other hand, looking at our findings from another perspective, the subtle eye movements abnormalities showed even in clinically “pure” ET could represent *additional neurological signs of uncertain significance*, which may fulfil current criteria for “soft signs” in the same way as other clinical features characterizing ET-P (i.e. impaired tandem gait, questionable dystonia, rest tremor) (Bhatia et al. 2018). On this light, our findings may represent a further challenge to the current tremor classification, supporting the ongoing controversies (Louis et al. 2020).

Finally, it should be noted that no significant clinical-instrumental correlations nor differences concerning oculomotor parameters based on the presence/absence of single clinical “soft signs” were found in tremor population. Due to the relatively small sample size, a type II error cannot be entirely ruled out, thus that larger studies are needed to further investigate current exploratory findings.

Beside the relatively small sample size and the cross-sectional study design, some other points such as the lack of functional neuroimaging or other instrumental approaches to explore cerebellar activity should also be considered as a limit in interpreting our data, thus that the present investigation must be intended as an exploratory study.

In conclusion, in the present study SWJ subtypes analysis as well as saccadic eye movement quantitative assessment in ET have been highlighted as possible tools for the disease characterization. Further studies with larger sample size and a more specific neuroimaging and instrumental support are needed in order to confirm our hypotheses as well as to better elucidate the role of our findings in ET phenotyping.

## Supplementary Information

Below is the link to the electronic supplementary material.

Supplementary Material 1

## Data Availability

the data that support the findings of this study are available from the corresponding author, upon reasonable request.
