# Supplementary material for: The spectrum of square wave jerks in essential tremor: an exploratory study
Source: J Neural Transm (Vienna). 2026 Mar 17;133(9):2917–24. doi: 10.1007/s00702-026-03134-y (PMC13687398; doi:10.1007/s00702-026-03134-y)
Supplement: Supplementary file 1 [file 702_2026_3134_MOESM1_ESM.docx]

**Figure S1.** Horizontal Main Sequence Linear Models in tremor population (ET-TP) and HCs.

**Table S1.** MSWJ quantitative parameters across study groups.

|  | **HCs**  **N=17** | **Tremor population**  **N=36** | **p-value** | **Adj p-value^§^** |
| --- | --- | --- | --- | --- |
| **S1 – Amplitude (deg)** | 0.85 ± 0.24 | 1.26 ± 1.13 | 0.179 | 0.191 |
| **S1 – Mean Velocity (deg/s)** | 50.56 ± 8.63 | 54.54 ± 16.36 | 0.617 | 0.498 |
| **S1 – Peak Velocity (deg/s)** | 78.78 ± 20.91 | 79.20 ± 45.45 | 0.799 | 0.550 |
| **S1 – Duration (ms)** | 19.71 ± 5.78 | 20.55 ± 12.10 | 0.356 | 0.915 |
| **ISI (ms)** | 190.79 ± 79.16 | 198.10 ± 68.61 | 0.828 | 0.536 |
| **S2 – Amplitude (deg)** | 0.71 ± 0.26 | 1.11 ± 1.08 | 0.246 | 0.224 |
| **S2 – Mean Velocity (deg/s)** | 47.62 ± 13.89 | 52.79 ± 19.00 | 0.320 | 0.474 |
| **S2 – Peak Velocity (deg/s)** | 71.84 ± 27.74 | 79.20 ± 45.45 | 0.902 | 0.779 |
| **S2 – Duration (ms)** | 17.64 ± 6.29 | 16.12 ± 7.01 | 0.309 | 0.326 |
|  | **Pure ET**  **N=8** | **ET-Plus**  **N=28** | **p-value** | **Adj p-value^§^** |
| **S1 – Amplitude (deg)** | 0.97 ± 0.25 | 1.35 ± 1.26 | 0.814 | 0.427 |
| **S1 – Mean Velocity (deg/s)** | 50.05 ± 6.47 | 55.82 ± 18.12 | 0.751 | 0.393 |
| **S1 – Peak Velocity (deg/s)** | 86.93 ± 49.10 | 85.05 ± 40.00 | 0.896 | 0.899 |
| **S1 – Duration (ms)** | 16.47 ± 2.51 | 21.72 ± 13.48 | 0.483 | 0.326 |
| **ISI (ms)** | 190.58 ± 76.75 | 200.25 ± 67.48 | 0.730 | 0.786 |
| **S2 – Amplitude (deg)** | 0.84 ± 0.30 | 1.19 ± 1.20 | 0.980 | 0.441 |
| **S2 – Mean Velocity (deg/s)** | 46.84 ± 7.72 | 54.49 ± 20.95 | 0.466 | 0.318 |
| **S2 – Peak Velocity (deg/s)** | 67.49 ± 16.23 | 82.55 ± 50.57 | 0.808 | 0.421 |
| **S2 – Duration (ms)** | 14.92 ± 3.86 | 16.46 ± 7.70 | 0.716 | 0.613 |

**Table S1 Legend.** ^§^: adjusted by age; S1: First saccadic intrusion; S2: Second saccadic intrusion; ISI: intersaccadic interval.

**Table S2.** BSWJ quantitative parameters across study groups.

|  | **HCs**  **N=2** | **Tremor population**  **N=18** | **p-value** | **Adj p-value^§^** |
| --- | --- | --- | --- | --- |
| **S1 – Amplitude (deg)** | 0.69 ± 0.23 | 0.94 ± 0.80 | 0.937 | 0.758 |
| **S1 – Mean Velocity (deg/s)** | 43.41 ± 3.51 | 49.47 ± 18.34 | 0.853 | 0.563 |
| **S1 – Peak Velocity (deg/s)** | 57.67 ± 5.60 | 68.40 ± 26.93 | 0.842 | 0.458 |
| **S1 – Duration (ms)** | 14.00 ± 1.41 | 15.90 ± 6.07 | 0.968 | 0.743 |
| **ISI 1 (ms)** | 74.25 ± 36.42 | 109.28 ± 53.21 | 0.432 | 1.000 |
| **S2 – Amplitude (deg)** | 1.36 ± 0.36 | 1.71 ± 0.93 | 0.747 | 0.683 |
| **S2 – Mean Velocity (deg/s)** | 68.98 ± 8.77 | 67.79 ± 17.57 | 0842 | 0.915 |
| **S2 – Peak Velocity (deg/s)** | 115.64 ± 50.59 | 117.62 ± 41.91 | 0.853 | 0.713 |
| **S2 – Duration (ms)** | 21.25 ± 8.84 | 23.80 ± 7.35 | 0.737 | 0.561 |
| **ISI 2 (ms)** | 173.75 ± 109.25 | 162.70 ± 56.69 | 1.000 | 1.000 |
| **S3 – Amplitude (deg)** | 0.95 ± 0.55 | 1.00 ± 0.43 | 0.937 | 0.267 |
| **S3 – Mean Velocity (deg/s)** | 48.56 ± 19.05 | 52.90 ± 12.70 | 0.758 | 0.307 |
| **S3 – Peak Velocity (deg/s)** | 88.26 ± 42.35 | 79.41 ± 32.19 | 0.747 | 0.770 |
| **S3 – Duration (ms)** | 20.25 ± 6.01 | 16.37 ± 5.18 | 0.358 | 0.539 |
|  | **Pure ET**  **N=6** | **ET-Plus**  **N=12** | **p-value** | **Adj p-value^§^** |
| **S1 – Amplitude (deg)** | 1.11 ± 1.11 | 0.85 ± 0.65 | 0.602 | 0.491 |
| **S1 – Mean Velocity (deg/s)** | 49.28 ± 16.79 | 49.57 ± 19.80 | 0.601 | 0.868 |
| **S1 – Peak Velocity (deg/s)** | 71.29 ± 34.66 | 66.95 ± 23.84 | 0.874 | 0.640 |
| **S1 – Duration (ms)** | 16.33 ± 7.30 | 15.69 ± 5.71 | 0.803 | 0.629 |
| **ISI 1 (ms)** | 124.82 ± 65.03 | 101.50 ± 47.49 | 0.481 | 0.428 |
| **S2 – Amplitude (deg)** | 1.64 ± 1.27 | 1.74 ± 0.77 | 0.373 | 0.968 |
| **S2 – Mean Velocity (deg/s)** | 65.10 ± 21.05 | 69.14 ± 16.43 | 0.479 | 0.874 |
| **S2 – Peak Velocity (deg/s)** | 106.98 ± 42.59 | 122.94 ± 42.40 | 0.280 | 0.665 |
| **S2 – Duration (ms)** | 21.74 ± 6.98 | 24.82 ± 7.61 | 0.599 | 0.643 |
| **ISI 2 (ms)** | 156.00 ± 50.50 | 166.05 ± 61.41 | 0.945 | 0.760 |
| **S3 – Amplitude (deg)** | 0.91 ± 0.30 | 1.04 ± 0.49 | 0.733 | 0.708 |
| **S3 – Mean Velocity (deg/s)** | 49.23 ± 8.09 | 54.73 ± 14.44 | 0.601 | 0.545 |
| **S3 – Peak Velocity (deg/s)** | 71.67 ± 18.58 | 82.29 ± 37.36 | 0.874 | 0.646 |
| **S3 – Duration (ms)** | 15.06 ± 3.64 | 17.03 ± 5.84 | 0.630 | 0.619 |

**Table S2 Legend.** ^§^: adjusted by age; S1: First saccadic intrusion; S2: Second saccadic intrusion; S3: Third saccadic intrusion; ISI 1: First intersaccadic interval; ISI 2: Second intersaccadic interval.

**Table S3.** SSWJ quantitative parameters across study groups.

|  | **Pure ET**  **N=3** | **ET-Plus**  **N=5** | **p-value** | **Adj p-value^§^** |
| --- | --- | --- | --- | --- |
| **Number of steps** | 2.0 ± 0.0 | 2.0 ± 0.0 | 1.000 | **/** |
| **S1 – Amplitude (deg)** | 1.8 ± 0.27 | 6.02 ± 3.09 | **0.036*** | / |
| **S1 – Mean Velocity (deg/s)** | 86.23 ± 22.01 | 121.89 ± 36.89 | 0.393 | / |
| **S1 – Peak Velocity (deg/s)** | 146.56 ± 40.81 | 191.81 ± 41.36 | 0.393 | / |
| **S1 – Duration (ms)** | 23.83 ± 2.75 | 47.2 ± 18.10 | 0.071 | / |
| **ISI 1 (ms)** | 161.33 ± 77.44 | 170.4 ± 66.13 | 0.786 | / |
| **S2 – Amplitude (deg)** | 1.43 ± 0.60 | 5.07 ± 2.85 | 0.143 | / |
| **S2 – Mean Velocity (deg/s)** | 67.07 ± 18.93 | 110.45 ± 43.97 | 0.393 | / |
| **S2 – Peak Velocity (deg/s)** | 102.66 ± 39.09 | 189.54 ± 65.46 | 0.143 | / |
| **S2 – Duration (ms)** | 18.83 ± 3.88 | 50.4 ± 21.50 | 0.071 | / |
| **ISI 2 (ms)** | 263.67 ± 70.50 | 198.6 ± 130.54 | 0.250 | / |
| **S3 – Amplitude (deg)** | 0.58 ± 0.05 | 0.91 ± 0.48 | 0.250 | / |
| **S3 – Mean Velocity (deg/s)** | 41.99 ± 2.47 | 43.46 ± 3.05 | 0.393 | / |
| **S3 – Peak Velocity (deg/s)** | 49.54 ± 2.22 | 63.39 ± 18.31 | 0.250 | / |
| **S3 – Duration (ms)** | 10.50 ± 0.50 | 19.80 ± 17.18 | 0.214 | / |

**Table S3 Legend.** ^§^: adjusted by age; S1: First saccadic intrusion; S2: Second saccadic intrusion; S3: Third saccadic intrusion; ISI 1: First intersaccadic interval; ISI 2: Second intersaccadic interval; *: p < 0.050.

**Table S4.** TSWJ quantitative parameters across study groups.

|  | **Pure ET**  **N=2** | **ET-Plus**  **N=3** | **p-value** | **Adj p-value^§^** |
| --- | --- | --- | --- | --- |
| **S1 – Amplitude (deg)** | 5.54 ± 6.79 | 1.04 ± 0.02 | 1.000 | / |
| **S1 – Mean Velocity (deg/s)** | 41.19 ± 4.12 | 50.11 ± 1.23 | 0.200 | / |
| **S1 – Peak Velocity (deg/s)** | 60.76 ± 9.02 | 76.48 ± 8.12 | 0.200 | / |
| **S1 – Duration (ms)** | 15.87 ± 1.24 | 19.17 ± 2.93 | 0.200 | / |
| **ISI 1 (ms)** | 75.12 ± 4.42 | 64.17 ± 0.29 | 0.200 | / |
| **S2 – Amplitude (deg)** | 1.09 ± 0.00 | 1.69 ± 0.00 | 0.200 | / |
| **S2 – Mean Velocity (deg/s)** | 54.66 ± 1.77 | 70.18 ± 2.51 | 0.200 | / |
| **S2 – Peak Velocity (deg/s)** | 57.55 ± 5.65 | 118.78 ± 5.88 | 0.200 | / |
| **S2 – Duration (ms)** | 18.25 ± 1.77 | 22.5 ± 0.5 | 0.200 | / |
| **ISI 2 (ms)** | 63.37 ± 40.48 | 79.17 ± 73.03 | 0.800 | / |
| **S3 – Amplitude (deg)** | 1.79 ± 0.84 | 2.64 ± 0.67 | 0.400 | / |
| **S3 – Mean Velocity (deg/s)** | 74.91 ± 26.08 | 93.88 ± 13.84 | 0.400 | / |
| **S3 – Peak Velocity (deg/s)** | 129.13 ± 44.56 | 158.25 ± 14.63 | 0.400 | / |
| **S3 – Duration (ms)** | 22.5 ± 2.12 | 27.33 ± 2.08 | 0.200 | / |
| **ISI 3 (ms)** | 130.0 ± 0.0 | 139.0 ± 77.94 | 0.600 | / |
| **S4 – Amplitude (deg)** | 0.82 ± 0.49 | 1.52 ± 0.61 | 0.400 | / |
| **S4 – Mean Velocity (deg/s)** | 47.99 ± 11.49 | 64.05 ± 17.62 | 0.400 | / |
| **S4 – Peak Velocity (deg/s)** | 66.17 ± 29.69 | 105.88 ± 36.01 | 0.400 | / |
| **S4 – Duration (ms)** | 13.12 ± 7.25 | 21.5 ± 3.5 | 0.400 | / |

**Table S4 Legend.** ^§^: adjusted by age; S1: First saccadic intrusion; S2: Second saccadic intrusion; S3: Third saccadic intrusion; S4: Fourth saccadic intrusion; ISI 1: First intersaccadic interval; ISI 2: Second intersaccadic interval; ISI 3: Third intersaccadic interval.

**Table S5.** Saccadic Parameters in Total Tremor Population vs HCs

|  | **HCs**  **N=23** | **Tremor population**  **N=40** | **p-value** | **Adj p-value^§^** |
| --- | --- | --- | --- | --- |
| **Horizontal Saccades 10 Deg** |  |  |  |  |
| Latency (ms) | 206.7 ± 33.0 | 236.1 ± 54.6 | **0.031*** | 0.149 |
| *Latency VC* | 0.18 ± 0.05 | 0.22 ± 0.10 | 0.144 | 0.290 |
| Amplitude (deg) | 9.6 ± 0.9 | 9.2 ± 1.1 | 0.158 | 0.143 |
| *Amplitude VC* | 0.09 ± 0.05 | 0.14 ± 0.07 | **0.006*** | 0.064 |
| Duration (ms) | 52.3 ± 6.6 | 55.7 ± 8.9 | 0.367 | 0.439 |
| *Duration VC* | 0.11 ± 0.05 | 0.14 ± 0.06 | 0.176 | 0.163 |
| Mean Velocity (deg/s) | 189.7 ± 18.0 | 177.4 ± 23.1 | **0.038*** | 0.095 |
| *Mean Velocity VC* | 0.10 ± 0.04 | 0.13 ± 0.05 | **0.031*** | 0.099 |
| Peak Velocity (deg/s) | 346.3 ± 12.1 | 313.6 ± 56.4 | **0.034*** | 0.147 |
| *Peak Velocity VC* | 0.11 ± 0.05 | 0.15 ± 0.08 | 0.148 | 0.133 |
| Gain | 0.94 ± 0.07 | 0.90 ± 0.10 | 0.088 | 0.204 |
| *Gain VC* | 0.10 ± 0.05 | 0.14 ± 0.07 | **0.015*** | 0.101 |
| **Horizontal Saccades 15 Deg** |  |  |  |  |
| Latency (ms) | 233.8 ± 43.2 | 269.4 ± 53.8 | **0.034*** | 0.233 |
| *Latency VC* | 0.20 ± 0.06 | 0.26 ± 0.08 | **0.006*** | 0.085 |
| Amplitude (deg) | 13.3 ± 0.6 | 12.9 ± 1.1 | 0.161 | 0.298 |
| *Amplitude VC* | 0.07 ± 0.03 | 0.14 ± 0.08 | **0.002*** | 0.057 |
| Duration (ms) | 63.9 ± 5.2 | 66.7 ± 7.7 | 0.216 | 0.391 |
| *Duration VC* | 0.10 ± 0.03 | 0.14 ± 0.07 | 0.097 | 0.151 |
| Mean Velocity (deg/s) | 219.6 ± 19.1 | 204.2 ± 27.5 | 0.063 | 0.193 |
| *Mean Velocity VC* | 0.09 ± 0.01 | 0.14 ± 0.06 | **0.003*** | **0.034*** |
| Peak Velocity (deg/s) | 384.4 ± 62.3 | 341.0 ± 62.2 | **0.034*** | 0.168 |
| *Peak Velocity VC* | 0.09 ± 0.03 | 0.17 ± 0.09 | **0.002*** | **0.026*** |
| Gain | 0.92 ± 0.05 | 0.87 ± 0.08 | **0.022*** | 0.063 |
| *Gain VC* | 0.08 ± 0.03 | 0.14 ± 0.08 | **0.001*** | 0.053 |
| **Vertical Saccades 5 Deg** |  |  |  |  |
| Latency (ms) | 226.9 ± 39.3 | 244.0 ± 36.0 | 0.094 | 0.316 |
| *Latency VC* | 0.19 ± 0.07 | 0.21 ± 0.08 | 0.411 | 0.884 |
| Amplitude (deg) | 4.32 ± 0.64 | 4.27 ± 0.61 | 0.775 | 0.502 |
| *Amplitude VC* | 0.21 ± 0.09 | 0.25 ± 0.10 | 0.140 | 0.524 |
| Duration (ms) | 37.7 ± 4.1 | 37.8 ± 6.1 | 0.582 | 0.820 |
| *Duration VC* | 0.18 ± 0.08 | 0.19 ± 0.09 | 0.378 | 0.836 |
| Mean Velocity (deg/s) | 120.1 ± 13.6 | 118.9 ± 14.2 | 0.753 | 0.570 |
| *Mean Velocity VC* | 0.17 ± 0.05 | 0.17 ± 0.05 | 0.863 | 0.478 |
| Peak Velocity (deg/s) | 218.3 ± 38.7 | 207.9 ± 42.5 | 0.353 | 0.834 |
| *Peak Velocity VC* | 0.21 ± 0.07 | 0.22 ± 0.09 | 0.886 | 0.836 |
| Gain | 0.96 ± 0.20 | 0.90 ± 0.16 | 0.174 | 0.828 |
| *Gain VC* | 0.23 ± 0.09 | 0.28 ± 0.12 | 0.165 | 0.380 |
| **Vertical Saccades 8 Deg** |  |  |  |  |
| Latency (ms) | 226.9 ± 40.5 | 259.8 ± 38.1 | **0.011*** | 0.104 |
| *Latency VC* | 0.18 ± 0.08 | 0.24 ± 0.15 | 0.071 | 0.307 |
| Amplitude (deg) | 6.7 ± 0.8 | 6.5 ± 0.8 | 0.478 | 0.733 |
| *Amplitude VC* | 0.20 ± 0.11 | 0.22 ± 0.08 | 0.483 | 0.533 |
| Duration (ms) | 46.5 ± 3.1 | 46.0 ± 5.5 | 0.336 | 0.993 |
| *Duration VC* | 0.17 ± 0.08 | 0.18 ± 0.06 | 0.817 | 0.276 |
| Mean Velocity (deg/s) | 151.5 ± 19.2 | 157.1 ± 55.2 | 0.527 | 0.922 |
| *Mean Velocity VC* | 0.14 ± 0.06 | 0.21 ± 0.31 | 0.199 | 0.475 |
| Peak Velocity (deg/s) | 279.2 ± 50.8 | 259.5 ± 45.8 | 0.199 | 0.605 |
| *Peak Velocity VC* | 0.18 ± 0.06 | 0.19 ± 0.08 | 0.642 | 0.587 |
| Gain | 0.90 ± 0.11 | 0.84 ± 0.12 | 0.119 | 0.425 |
| *Gain VC* | 0.22 ± 0.11 | 0.24 ± 0.08 | 0.505 | 0.530 |

**Table S5 Legend.** ^§^: adjusted by age; *: p < 0.050.

**Table S6.** Saccadic Parameters in ET vs ET-Plus

|  | **Pure ET**  **N=10** | **ET-Plus**  **N=26** | **p-value** | **Adj p-value^§^** |
| --- | --- | --- | --- | --- |
| **Horizontal Saccades 10 Deg** |  |  |  |  |
| Latency (ms) | 236.6 ± 72.7 | 235.9 ± 48.7 | 0.818 | 0.920 |
| *Latency VC* | 0.22 ± 0.09 | 0.23 ± 0.10 | 0.842 | 0.854 |
| Amplitude (deg) | 9.4 ± 1.3 | 9.2 ± 1.0 | 0.672 | 0.623 |
| *Amplitude VC* | 0.16 ± 0.08 | 0.13 ± 0.07 | 0.315 | 0.230 |
| Duration (ms) | 55.7 ± 14.1 | 55.3 ± 6.7 | 0.569 | 0.647 |
| *Duration VC* | 0.13 ± 0.06 | 0.14 ± 0.07 | 0.914 | 0.853 |
| Mean Velocity (deg/s) | 178.3 ± 27.4 | 177.1 ± 22.1 | 0.890 | 0.894 |
| *Mean Velocity VC* | 0.15 ± 0.07 | 0.12 ± 0.05 | 0.172 | 0.175 |
| Peak Velocity (deg/s) | 316.6 ± 42.0 | 312.7 ± 61.0 | 0.852 | 0.869 |
| *Peak Velocity VC* | 0.20 ± 0.12 | 0.13 ± 0.06 | 0.116 | **0.047*** |
| Gain | 0.90 ± 0.10 | 0.89 ± 0.11 | 0.878 | 0.873 |
| *Gain VC* | 0.17 ± 0.08 | 0.13 ± 0.07 | 0.156 | 0.153 |
| **Horizontal Saccades 15 Deg** |  |  |  |  |
| Latency (ms) | 266.3 ± 66.5 | 270.2 ± 51.4 | 0.865 | 0.976 |
| *Latency VC* | 0.24 ± 0.09 | 0.26 ± 0.08 | 0.519 | 0.619 |
| Amplitude (deg) | 13.1 ± 0.7 | 12.8 ± 1.2 | 0.564 | 0.688 |
| *Amplitude VC* | 0.13 ± 0.05 | 0.14 ± 0.09 | 1.000 | 0.939 |
| Duration (ms) | 69.8 ± 9.2 | 65.9 ± 7.3 | 0.890 | 0.113 |
| *Duration VC* | 0.14 ± 0.06 | 0.14 ± 0.07 | 0.050 | 0.633 |
| Mean Velocity (deg/s) | 201.7 ± 20.8 | 204.8 ± 29.4 | 0.797 | 0.502 |
| *Mean Velocity VC* | 0.17 ± 0.07 | 0.14 ± 0.06 | 0.346 | 0.154 |
| Peak Velocity (deg/s) | 347.3 ± 50.0 | 339.3 ± 65.8 | 0.768 | 0.981 |
| *Peak Velocity VC* | 0.20 ± 0.10 | 0.16 ± 0.08 | 0.123 | 0.238 |
| Gain | 0.88 ± 0.06 | 0.87 ± 0.09 | 0.625 | 0.716 |
| *Gain VC* | 0.14 ± 0.05 | 0.15 ± 0.09 | 0.594 | 0.965 |
| **Vertical Saccades 5 Deg** |  |  |  |  |
| Latency (ms) | 238.6 ± 40.8 | 243.6 ± 34.6 | 0.656 | 0.606 |
| *Latency VC* | 0.21 ± 0.11 | 0.21 ± 0.07 | 0.488 | 0.849 |
| Amplitude (deg) | 4.3 ± 0.6 | 4.3 ± 0.6 | 0.984 | 0.910 |
| *Amplitude VC* | 0.27 ± 0.07 | 0.25 ± 0.11 | 0.613 | 0.459 |
| Duration (ms) | 37.0 ± 2.8 | 38.3 ± 7.2 | 0.747 | 0.619 |
| *Duration VC* | 0.21 ± 0.05 | 0.19 ± 0.10 | 0.450 | 0.275 |
| Mean Velocity (deg/s) | 117.0 ± 14.4 | 119.2 ± 14.5 | 0.714 | 0.607 |
| *Mean Velocity VC* | 0.17 ± 0.04 | 0.17 ± 0.06 | 0.747 | 0.544 |
| Peak Velocity (deg/s) | 197.5 ± 30.8 | 212.3 ± 46.8 | 0.366 | 0.374 |
| *Peak Velocity VC* | 0.24 ± 0.06 | 0.22 ± 0.10 | 0.608 | 0.417 |
| Gain | 0.89 ± 0.17 | 0.88 ± 0.14 | 0.808 | 0.893 |
| *Gain VC* | 0.31 ± 0.14 | 0.28 ± 0.11 | 0.401 | 0.323 |
| **Vertical Saccades 8 Deg** |  |  |  |  |
| Latency (ms) | 273.1 ± 54.2 | 250.2 ± 30.4 | 0.166 | 0.304 |
| *Latency VC* | 0.20 ± 0.04 | 0.22 ± 0.08 | 0.563 | 0.541 |
| Amplitude (deg) | 6.7 ± 0.7 | 6.5 ± 0.9 | 0.674 | 0.547 |
| *Amplitude VC* | 0.25 ± 0.09 | 0.22 ± 0.08 | 0.361 | 0.141 |
| Duration (ms) | 47.4 ± 7.3 | 46.0 ± 5.2 | 0.570 | 0.556 |
| *Duration VC* | 0.19 ± 0.06 | 0.18 ± 0.06 | 0.556 | 0.266 |
| Mean Velocity (deg/s) | 148.0 ± 15.4 | 161.6 ± 66.7 | 0.601 | 0.820 |
| *Mean Velocity VC* | 0.17 ± 0.08 | 0.23 ± 0.38 | 0.672 | 0.761 |
| Peak Velocity (deg/s) | 254.0 ± 25.1 | 261.7 ± 51.6 | 0.707 | 0.631 |
| *Peak Velocity VC* | 0.23 ± 0.09 | 0.19 ± 0.07 | 0.170 | 0.197 |
| Gain | 0.85 ± 0.10 | 0.83 ± 0.12 | 0.612 | 0.711 |
| *Gain VC* | 0.27 ± 0.08 | 0.23 ± 0.09 | 0.291 | 0.111 |

**Table S6 Legend.** ^§^: adjusted by age; *: p < 0.050.
